# Early life adverse environmental, nutrition and infection factors are associated with lower developmental scores in Pakistani children at 5 years: a cohort study

**DOI:** 10.1136/bmjnph-2024-000900

**Published:** 2024-11-07

**Authors:** Doris González-Fernández, Aisha Yousafzai, Simon Cousens, Arjumand Rizvi, Imran Ahmed, Sajid Bashir Soofi, Zulfiqar Ahmed Bhutta

**Affiliations:** 1 Centre for Global Child Health, The Hospital for Sick Children, Toronto, Ontario, Canada; 2 Department of Global Health and Population, Harvard University T H Chan School of Public Health, Boston, Massachusetts, USA; 3 Department of Infectious Disease Epidemiology, London School of Hygiene & Tropical Medicine, London, UK; 4 Aga Khan University, Karachi, Pakistan; 5 Pediatrics & Child Health, Aga Khan University, Karachi, Pakistan; 6 Center of Excellence in Women & Child Health, Aga Khan University, Karachi, Pakistan; 7 Institute for Global Health and Development, Aga Khan University, Karachi, Pakistan

**Keywords:** Cognitive performance, Infectious disease

## Abstract

**Background:**

The effects of multiple early adverse psychosocial and biological factors on child development at preschool age in deprived settings are not fully understood.

**Methods:**

The ‘Etiology, Risk Factors and Interactions of Enteric Infections and Malnutrition and the Consequences for Child Health and Development’ (MAL-ED) project followed children from eight countries, recording sociodemographic, nutritional, illness, enteroinfection biomarkers and scores for quality of home environment (Home Observation for Measurement of the Environment (HOME)), development (Bayley) and maternal depression during the first year of life. In the Pakistan cohort, we investigated associations of these early factors with Z-scores (derived from the eight participating countries) of three developmental outcomes at 5 years: Executive Functions (Z-EF), the Wechsler Preschool and Primary Scale for Intelligence (Z-WPPSI) and the externalising behaviours component of the Strength and Difficulties test (Z-externalising behaviours).

**Results:**

Most children had 5-year development measurements below other MAL-ED countries (Z-EF<0, 80.3%, Z-WPPSI<0, 69.3%) and 45.6% had Z-externalising behaviours>0. Higher Z-EF was associated with higher HOME (coeff: 0.03 (95% CI 0.005, 0.05), p=0.017) and Bayley scores (0.01 (0.002, 0.01), p=0.010). Higher Z-WPPSI was associated with more household assets (0.02 (0.01, 0.03), p=0.003), but with lower alpha-1 antitrypsin (µmol/L, protein-losing enteropathy) (−0.01 (−0.02, –0.005), p=0.003). Lower externalising behaviour was associated with female sex (−0.30 (−0.53, –0.08), p=0.009), higher soluble-transferrin-receptors (mg/L) (−0.07 (−0.14, –0.01), p=0.024) and initiation of solids/semisolids≥6 months (−0.16 (−0.31, –0.01), p=0.033), but higher externalising behaviour was associated with underweight (0.35 (0.07, 0.62), p=0.014), more diarrhoeal episodes (0.03 (0.004, 0.06), p=0.022) and higher Maternal Depression Score (0.04 (0.01, 0.07), p=0.003) in the first year.

**Conclusion:**

Adverse environmental, nutrition and infectious factors, and indicators of deprived early development in the first year of life have a negative association with developmental scores at 5 years. Addressing early stressors, improving diet, infections and environment stimulation early in life could positively impact child development in resource-constrained settings.

**WHAT IS ALREADY KNOWN ON THIS TOPIC**

- Early child development can be affected by early stressors in life, and several measurements have been developed for the evaluation of child intelligence and behaviour in developed settings. However, developmental evaluation is challenging in resource-constrained settings, where multiple adverse factors coexist.

**WHAT THIS STUDY ADDS**

- In a cohort of children followed from birth to 5 years, three developmental tests (Executive Functions, Wechsler Preschool and Primary Scale for Intelligence and the externalising behaviours component of the Strength and Difficulties test (Z-externalising behaviours)) were contextually adapted, and their association with multiple stressors during the first year of life (sociodemographic, nutritional and infectious indicators) were investigated.

**HOW THIS STUDY MIGHT AFFECT RESEARCH, PRACTICE OR POLICY**

- This study validates the use of developmental tools in resource-limited environments. Associations of child development at 5 years with exposure to poverty, poor nutrition and infections in the first year of life, highlight the need for early interventions to improve child development in resource-constrained settings.

## Introduction

 Child development, defined as the growth of physical, cognitive, psychological and socioemotional skills that allow competence, autonomy and independence,^1^ is a process that starts in utero. Around one-quarter of overall brain development occurs prenatally, evolving to two-thirds of the size of the adult brain by 3 years of age, when neural connections proliferate in areas of the brain associated with functions such as binocular vision, emotional control, language and cognitive abilities.^2^ Therefore, multiple adverse childhood experiences including environmental/sociodemographic and perinatal stressors, early malnutrition, inadequate stimulation and infectious diseases can affect biological mechanisms that influence child development.^3^

Tracking early child development (ECD) through population-based measurements for developmental outcomes is a global concern. The Early Childhood Development Index that evaluates child development of 3 and 4 years old was introduced to the Multiple Indicator Cluster Surveys (MICS) in 2009.^4^ Using MICS data, the prevalence of significant cognitive delay has been estimated in 2.6% in upper middle-income countries, 10.6% in lower middle-income countries (LMICs) and 20.7% in low-income countries.^5^ More recently, this measure has been expanded by increasing the number of developmental milestones and by including children from 2 years of age.^6^ Additionally, the WHO has introduced the Global Scales of Early Child Development that captures development in children from birth to 2 years.^7^ These measurements help understanding factors contributing to development outcomes at national and subnational level, but evaluating ECD is challenging in low-resource settings, as most development tests have been elaborated in upper-income countries.

The ‘Etiology, Risk Factors and Interactions of Enteric Infections and Malnutrition and the Consequences for Child Health and Development’—MAL-ED longitudinal birth cohort, followed children from eight LMICs (Pakistan, Bangladesh, India, Nepal, South Africa, Tanzania, Brazil and Peru), from birth to 5 years,^8^ and adapted a series of instruments to evaluate ECD. Among these instruments are the Executive Functions (EF) test, which evaluates cognitive skills for self-regulated attention, behaviour and emotions^9^; the Wechsler Preschool and Primary Scale-Third Edition (WPPSI-III), that provides an estimate of intellectual quotient and composite scores for specific domains of intelligence^10^; and the Strengths and Difficulties Questionnaire (SDQ), widely used for screening psychosocial problems, evaluates problem behaviour and competencies.^11^

In Pakistan, developmental delay has been estimated to be as high as 30% in the poorest children and has been associated with undernutrition and maternal depression.^12^ For this secondary analysis of the MAL-ED data from Pakistan, we aimed to investigate predictors of child development at 5 years assessed by EF, WPPSI and SDQ assessments, among factors during the first year of life, including Bayley scores, Maternal Depression Scores (using a Self-Reporting Questionnaire—SRQ), the Home Observation for Measurement of the Environment (HOME) inventory score, as well as indicators of child growth, sociodemographic characteristics, nutritional indicators, history of illness and environmental enteropathy markers.

## Methods

The MAL-ED cohort of Pakistan followed children from birth to 5 years of age from the Naushahro Feroze district in the Sindh province between November 2009 and February 2017.^13^ Developmental assessments were performed to evaluate (a) behavioural control/cognitive flexibility, using EF, (b) intelligence, using WPPSI-III and (c) behavioural problems, using SDQ.

**Figure 1:**
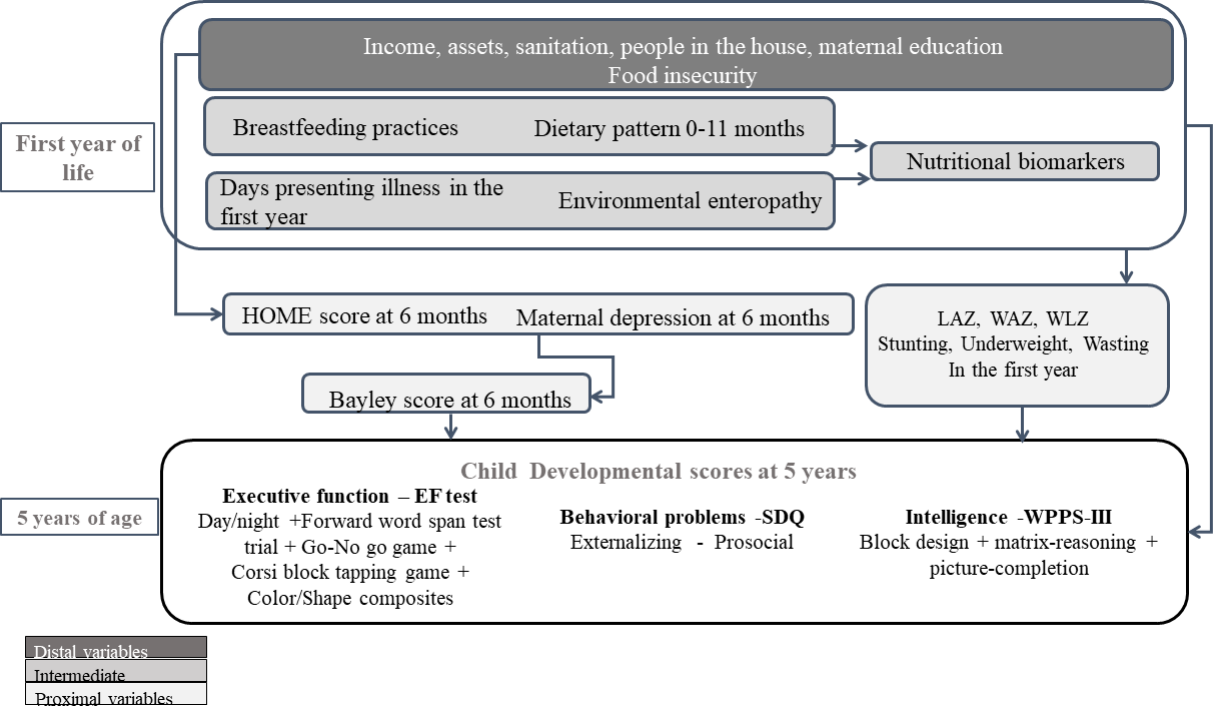
Conceptual framework, showing possible paths of early stressors (during the first year of life) classified as distal, intermediate and proximal factors, influencing child development at 5 years. EF, Executive Functions; HOME, Home Observation for Measurement of the Environment; LAZ, length for age Z-scores; SDQ, Strengths and Difficulties Questionnaire; WAZ, weight for age Z-scores; WPPS-III, Wechsler Preschool and Primary Scale-Third Edition; WLZ, weight for length Z-scores.

A conceptual framework based on available information and previous MAL-ED work was developed, with possible predictors of child development at 5 years classified as distal, intermediate and proximal factors recorded between 0 and 11 months of age (figure 1).

### Outcome variables

The MAL-ED project made language, cultural and familiarity/recognisability adaptations to the instruments, together with a strict quality assurance/quality control protocol to guarantee the integrity of the data across sites.^14^

Subscores from the EF, WPPSI-III and SDQ were converted into Z-scores using information from MAL-ED countries where data was considered reliable and are used as standard for the present study. India was excluded from the SDQ analyses, and Nepal from the EF analyses; Pakistan was retained in all cases. Quality control of data collection and analysis is detailed in online supplemental table S1.

#### EF Z-scores

Adaptations of the EF test were developed for another study in Pakistan,^9^ and used the following subscales (a) *Day-Night Stroop* test: evaluates interference control; (b) *Word Span test*: evaluates working memory; (c) *Go-no Go trial*: evaluates inhibitory control; (d) *Corsi block-tapping task*: evaluates visual-spatial working memory; (e) *Dimensional Change Card Sort*: evaluates cognitive flexibility. Composite scores were created for each of the subscales by adding the total number of correct items.

#### WPPSI-III Z-scores

The WPPSI-III for 4 to 7-year-old children includes performance, verbal and general language and processing speed composites. The performance score was used for the MAL-ED study, which encompasses block design, matrix reasoning and picture concept, measuring fluid reasoning, spatial processing skills, attentiveness to detail and visual-motor coordination skills.^10^ For these scores, more than 90% of WPPSI-III items were adapted, including translation, redrawing of pictures to match cultural norms, addition of practice items and adaptations to item administration number and means. Therefore, scores derived from the study are not comparable to traditional versions of the WPPSI-III.

#### SDQ Z-scores

The SDQ evaluates positive and negative attributes using 25 items divided into 5 scales of 5 items each.^11^ Respondents were asked whether the item was not true (coded as 0), somewhat true (coded as 1) or certainly true (coded as 2). Given low reliability and a high rate of differential item functioning for most items of internalising symptoms and prosocial factors, we kept only externalising behaviour as our outcome variable, which included positive answers to the following questions: being restless, overactive, cannot stay still for long; constantly fidgeting or squirming; easily distracted, concentration wanders; not good attention span; often loses temper; often fights with other children or bullies them; often lies or cheats; steals from home, school or elsewhere.

### Independent variables recorded during the first year of life

Independent variables grouped as distal, intermediate and proximal determinants of child development are detailed in online supplemental table S2.

#### Distal and intermediate predictors

Methodological information about sociodemographic/environmental factors,^15^ feeding practices and nutritional biomarkers,^16^ illness,^17^ enteropathogens,^18^ and indicators of environmental enteropathy^19^ and child growth^20^ have been reported before, and are summarised in online supplemental table S2.

#### Proximal predictors recorded at 6 months of age

The Bayley Scales of Infant and Toddler Development (BSID-III) was adapted^21^ and used to determine infant development. The HOME inventory, previously validated within this sample,^22^ was used to evaluate the quality of the home environment; and the Maternal Depression Score, recorded using the SRQ and previously validated in the MAL-ED cohort,^23^ was used to assess depression and psychological disturbance in mothers. Details of proximal predictor variables can be found in online supplemental table S2.

### Statistical analyses

Analyses were run using STATA V.16. Descriptive statistics of developmental outcomes are presented as means±SD, and independent variables are presented as proportions and median/IQR for binary and continuous variables, respectively.

We used WHO’s indicators for assessing infant and young child feeding practices that include calculated variables on exclusive or mixed breastfeeding, the minimal diet diversity (≥4 average food groups from 6 to 11 months) and the minimal feed frequency (2–3 times/day for breastfed infants 6–8 months, and 3–4 times/day in breastfed children 9–23 months)^24^ (online supplemental table S2). A minimum acceptable diet was considered when both diversity and frequency were above minimal.^24^ Given that <10% of children had initiated solids/semisolids as recommended (after 6 months), the variable was categorised as below or above the IQR of food initiation in the population (3–5 months). For analysing intestinal infections, individual pathogens isolated from stool samples during the first year were classified as positive for virus, bacteria or protozoa, and were classified according with their coexistence with diarrhoeal episodes as absent, present without diarrhoea and present with diarrhoea (online supplemental table S2). Binary independent variables from all groups were considered for analysis if at least 10% of children were exposed.

Multivariable linear regression models were run for Z-EF, Z-WPPSI and Z-externalising behaviour in a hierarchical step-forward fashion, controlling for sex and retaining independent variables with p values <0.20. Following our conceptual framework (figure 1), we started by adding distal predictors (sociodemographic characteristics and food insecurity), followed by one-by-one each of the six groups of intermediate predictors (breastfeeding and complementary feeding practices, illness, enteropathogens, faecal biomarkers, anthropometry and biomarkers of nutritional and inflammation status) recorded during the first year of age. Finally, direct predictors recorded at 6 months of age (Bayley subscores and total scores recorded at 6 and 15 months, HOME subscores and total scores, and maternal depression total score), were added to the models. Correlated variables of the same group were evaluated separately. Missing data was not inputted, and complete case analyses were run. We confirmed that outcome variables were jointly missing completely at random and that they jointly had covariate-dependent missingness.^25^ Models were checked for regression assumptions including collinearity using a variance inflation factor <10, heteroscedasticity (Breusch-Pagan test p>0.05), normality of residuals (Shapiro-Wilk W normality test p>0.01), influential observations (Cook’s distance p>1.00), specification problem using Linktest and appropriate functional form.

## Results

**Figure 2:**
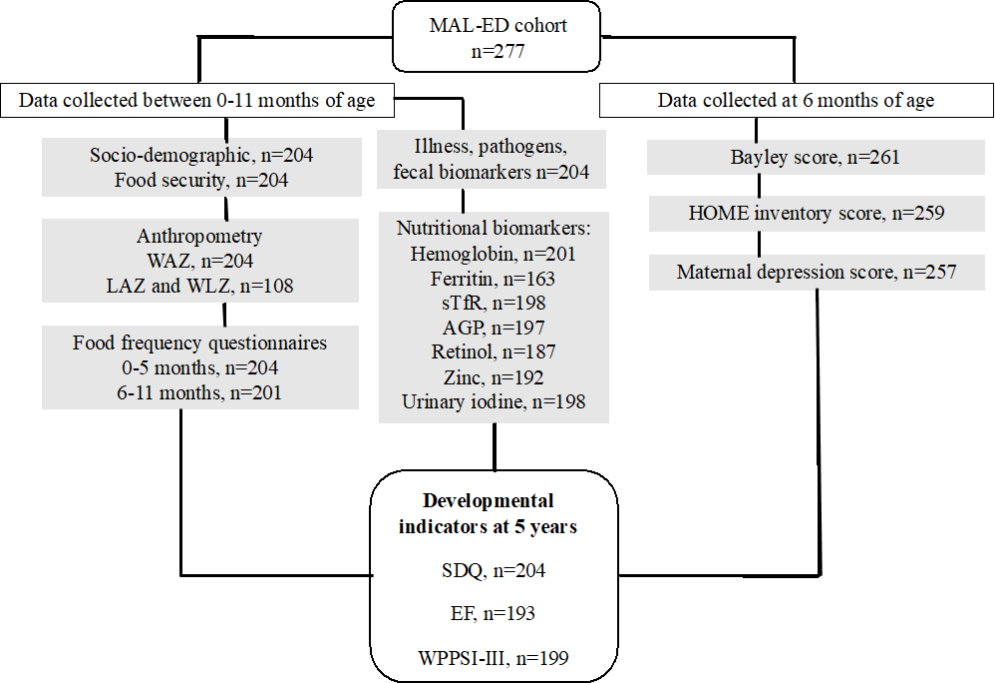
Study flowchart showing total number of children in the cohort, and sample size for data collected during the first year of life and for developmental outcomes at 5 years. HOME, Home Observation for Measurement of the Environment; LAZ, length for age Z-scores; MAL-ED, Etiology, Risk Factors and Interactions of Enteric Infections and Malnutrition and the Consequences for Child Health and Development; SDQ, Strengths and Difficulties Questionnaire; WAZ, weight for age Z-scores; WPPS-III, Wechsler Preschool and Primary Scale-Third Edition; WLZ, weight for length Z-scores.

As shown in the study flowchart (figure 2), of the 277 children of the cohort, 193 had EF data, 199 had WPPSI data and 204 had SDQ data at 5 years of age. Among groups of covariates, we found missing completely at random data for anthropometry (height data n=108), nutritional and inflammation biomarkers (n=163–201). Population characteristics are shown in table 1. Families of children in the study lived in poverty, with a median number of assets of three out of eight (detailed in online supplemental table S2), with a median monthly income of 13 200 rupees/month (current ~US$160), and 73% lived with some degree of food insecurity. Almost half of the mothers had not received any education, most households had improved sanitation and hosted a median of 3 people per room (table 1A).

**Table 1:** Sociodemographic characteristics, food insecurity and nutritional indicators*

	n (%)	Median (IQR)
**(A)Sociodemographic and food insecurity**		
Number of assets (range: 0–8)		3 (1, 4.5)
Income (rupees ×10^3^)		13.2 (8.0, 20.0)
Maternal education (years)		2 (0, 5)
Non-educated mothers	96 (47.1%)	
Improved sanitation	164 (80.4%)	
People per room		3 (2, 5)
Food insecurity category		
No food insecurity	55 (27.0%)	
Mild	63 (30.9%)	
Moderate/severe	86 (42.1%)	
**(B)Breastfeeding practices**		
Breastfeed 0–11 months	204 (100%)	
Exclusive BF throughout the first 6 months	0	
Days of exclusive BF in the first 6 months		14 (8–18)
Mixed milk feeding under 6 months	204 (100%)	
Continued breastfeeding 6–11 months	191 (94.1%)	
Introduction of solid/semisolid foods 0–5 months	190 (93.1%)	
**(C)Complementary feeding practices**		
Mean age (months) of solid/semisolids introduction		3.5 (1.8, 4.8)
Consumed solid/semisolid foods 6–8 months	192 (94.1%)	
Number of food groups intake between 6 and 11 months		3 (range: 1–6)
Minimum dietary diversity 6–11 months (n=181)†	7 (3.9%)	
Minimum meal frequency 6–11 months (n=203)‡	40 (19.7%)	
Minimum acceptable diet 6–11 months (n=181)§	2 (1.1%)	
Egg and/or flesh food consumption 6–11 months (n=184)	32 (17.4%)	
Unhealthy food consumption 6–11 months (n=204)¶	68 (33.3%)	
Zero vegetable or fruit consumption 6–11 months (n=204)	175 (85.8%)	
**(D)Infant anthropometry0–11months**		
Weight for age Z-scores		−1.53 (–2.15, –0.89)
Underweight	49 (24.0%)	
Length for age Z-scores (n=108)		−1.70 (–2.35, –1.10)
Stunting	26 (24.1%)	
Weight for length Z-scores (n=108)		−0.80 (–1.38, –0.25)
Wasting	11 (10.2%)	
Head circumference for age, Z-scores (n=108)		−0.96 (–1.52, –0.27)
Low head circumference	11 (10.2%)	
**(E)Infant anthropometry54–66months**		
Weight for age Z-scores (n=203)		−1.86 (–2.39, –1.26)
Underweight	86 (42.4%)	
Length for age Z-scores (n=108)		−2.06 (–2.67, –1.40)
Stunting	109 (54.0%)	
Weight for length Z-scores(n=108)		−0.66 (–1.26, –0.15)
Wasting	12 (5.9%)	

We report average values for: (A) 1.6 interviews, (B) daily breastfeeding evaluation, (C) monthly Food Frequency Questionnaire (FFQ), (D) three FFQ between 54 and 66 months, (E) monthly anthropometry measurements during the first year and two measurements between 54 and 66 months.

Definitions according to WHO guidelines for complementary feeding are explained in notes † to ¶.^36^

* Sample of 204 children unless otherwise specified.

† Consumed foods/beverages from at least five out of eight food groups

‡ Breastfed infants aged 6–8 months should be provided complementary foods 2–3 times per day and breastfed children aged 9–23 months should be provided complementary foods 3–4 times per day with additional nutritious snacks offered 1–2 times per day.

§ *For breastfed children*: receiving at least the minimum dietary diversity and minimum meal frequency for their age during the previous day; *for non-breastfed children*: receiving at least the minimum dietary diversity and minimum meal frequency for their age during the previous day as well as at least two milk feeds.

¶ Unhealthy foods refer to ‘sentinel unhealthy foods’ that are foods or categories of foods (eg, ‘sweets’ or ‘candies’) that are likely to be consumed by infants/young children and are high in sugar, salt and/or unhealthy fats.

Breastfeeding practices are shown in table 1B. Children received a median of 14 days of exclusive breastfeeding during their first 6 months of age (IQR=8–18 days), and although no children received exclusive BF throughout the first 6 months, BF was provided throughout the first year. During the 24-hour recall of the first month of age, 46% of babies had received water, 5% tea, 42% animal milk and 8% formula. Animal milk/formula intake started at a median age of 1 month (IQR=0.5, 2.4 months), and an early introduction of solid/semisolid foods happened at a median age of 3.5 months (IQR=1.8, 4.8 months) (table 1C). Out of the eight food groups as per WHO’s classification, children 6–11 months consumed a median of three groups (range: 1–6), with breastmilk and sweets the most commonly consumed. Reported intake of animal-source foods, fruits and vegetables in the first year of life was less than 10%. Average of 24 hours recalls showed that only 3.9% children had consumed the minimum dietary diversity, 19.7% had consumed the minimum meal frequency between 6 and 11 months of age, and only two children had consumed the minimum acceptable diet on at least one occasion during the previous day of the interviews.

The percentage of abnormal blood and urine biomarkers is shown in online supplemental table S3A. Children had high prevalence of deficiencies, notably anaemia (70.6%), zinc (83.8%) and vitamin A (54.0%) deficiencies. Iron deficiency indicated by low ferritin was found in 38.6%, but sTfR was elevated only in 5.6%. The burden of diseases during the first year was also important (online supplemental table S3B), children spending a median of 24 days/month with any type of illness, with evidence of environmental enteropathy (online supplemental table S3C) and intestinal infections comprising coexisting viruses, bacteria and protozoa (online supplemental table S3D).

Bayley developmental scores for motor, language and social development at 6 and 15 months, HOME inventory subscores and total scores, and Maternal Depression Scores recorded at 6 months are shown in table 2. At 5 years of age, most children had EF (80.3%) and WPPSI Z-scores (69.3%) below zero Z-scores, and almost half of the children (45.6%) showed externalising behaviour above zero Z-scores compared with other MAL-ED countries (table 2 and online supplemental figure S1).

**Table 2:** Developmental outcomes recorded at 5 years, and Bayley scores, Home Observation for Measurement of the Environment (HOME) and Maternal Depression Score (Strengths and Difficulties Questionnaire), recorded at 6 months of age

	n or %	Mean±SD	Median (IQR)
**Bayley score at 6months**	202	136.4±17.1	138 (128, 148)
Cognitive subscale		26.1±3.8	26 (24, 29)
Language (expressive+receptive) subscale		14.5±4.8	15 (10, 18)
Motor (fine+gross) subscale		38.7±7.0	40 (35, 43)
**Bayley score at 15months**	204		
Cognitive subscale		40.8±4.9	40 (38, 44)
Language (expressive+receptive) subscale		25.7±4.8	26 (23, 28)
Motor (fine+gross) subscale		70.0±6.2	70 (65, 75)
**HOME score (quality of home environment**)	201	32±6	32 (27, 37)
Emotional and verbal responsivity of caregiver		7.2±2.6	7 (5, 9)
Avoidance of restriction and punishment		3.4±2.3	5 (5, 5)
Promoting of child development		3.8±1.1	4 (3, 5)
Organisation of physical and temporal environment		7.6±2.9	8 (5, 10)
Provision of appropriate play materials		0.8±0.8	1 (0, 1)
Opportunities for variety in daily stimulation		6.4±1.3	6 (5, 7)
Cleanliness of child		2.7±0.8	3 (3, 3)
**SRQ (Maternal Depression Score, over 16 possible points**)	199	5.6±4.0	5 (2, 8)
**Developmental outcomes at 5years**			
Z-Executive Function (Z-EF)	193	−0.76±0.82	−0.80 (–1.38, –0.35)
Z-EF<0	80.3%		
Z-Wechsler Preschool and Primary Scale of Intelligence, III edition (Z-WPPSI)	199	−0.10±0.18	−0.12 (−0.25, 0.01)
Z-WPPSI<0	69.3%		
Z-externalising behaviour	204	−0.26±0.87	−0.23 (–1.06, 0.32)
Z-externalising behaviour>0	45.6%		

SRQSelf-Reporting Questionnaire

Final regression models for developmental indicators at 5 years, showed that EF Z-scores (adjusted R^2^=0.15) (table 3A), had positive associations with the HOME score at 6 months and the Bayley score measured at 15 months, but only weak evidence of an association with Maternal Depression Score at 6 months. We did not find evidence of associations between EF Z-scores and sex, micronutrient status, or intestinal infections during the first year.

**Table 3:** Linear regression models for Z-scores of developmental outcomes at 5 years: (A) Executive Functions (Z-EF), (B) Wechsler Preschool and Primary Scale of Intelligence-third edition (Z-WPPSI), (C) externalising behaviour and early indicators of infant’s health recorded during the first year of life

	Coefficient	95% CI	P value
**Z-EF**			
Sex (0: boys, 1: girls)	0.10	−0.13, 0.32	0.390
Food insecurity category(0: none, 1: mild, 2: moderate-severe)	−0.06	−0.22, 0.10	0.463
Mean days/month being ill 0–11 months	0.00	−0.02, 0.02	0.710
HOME inventory total score at 6 months	0.03	0.005, 0.05	0.017
Maternal SRQ at 6 months	−0.03	−0.06. 0.01	0.105
Total Bayley score at 15 months	0.01	0.002, 0.01	0.010
Constant	−2.77	−4.28, –1.26	<0.0001
**Z-WPPSI**			
Sex (boys: 0, girls: 1)	0.01	−0.04, 0.06	0.648
Assets number (0–8)	0.02	0.01, 0.03	0.003
Days presenting ALRI, 0–11 months	−0.04	−0.09, 0.02	0.197
Mean alpha-1 antitrypsin, µmol/L (0–11 months)	−0.01	−0.02, –0.005	0.003
Underweight 0–11 months	−0.01	−0.02, 0.002	0.103
Urinary iodine <100 µg/L, 0–11 months	−0.06	−0.13, 0.01	0.097
HOME subscale on ‘opportunities for variety in daily stimulation’	0.02	−0.001, 0.04	0.060
Constant	−0.19	−0.32, –0.05	0.006
**Z-externalisingbehaviour**			
Sex (0: boys, 1: girls)	−0.30	−0.53, –0.08	0.009
Age (months) starting solid/semisolids (0: 0–2, 1: 3–5, 2: 6–8)	−0.16	−0.31, –0.01	0.033
Underweight 0–11 months	0.35	0.07, 0.62	0.014
Number of diarrhoeal episodes 0–11 months	0.03	0.004, 0.06	0.022
sTfR, mg/L 0–11 months	−0.07	−0.14, –0.01	0.024
Bayley score at 6 months	0.006	−0.001, 0.01	0.075
Maternal SRQ at 6 months	0.04	0.01, 0.07	0.003
Constant	−1.13	−2.28, 0.02	0.055

A.(A) Model n=188, pp<0.0001, adj. R2=0.151, VIF=1.15, Breush-Pagan test pp=0.303.

B. (B) Model n=191, pp<0.0001, adj. R2=0.136, VIF=1.06, Breush-Pagan test pp=0.162.

C.(C) Model n=191, pp<0.0001, adj. R2=0.161, VIF=1.02, Breush-Pagan test pp=0.180.

ALRIacute lower respiratory infectionsHOMEHome Observation for Measurement of the EnvironmentSRQSelf-Reporting QuestionnaireVIFvariance inflation factor

The model for WPPSI Z-scores at 5 years, which explained 14% of the variability, evidenced a positive association with higher number of assets at home, whereas intestinal protein loss indicated by higher alpha-1 antitrypsin (AAT) between 0 and 11 months was a negative predictor. Days presenting acute low respiratory infection, underweight and iodine deficiency in the first year of life, as well as one of the subscales of the HOME score (opportunities for variety in daily stimulation) at 6 months had weak evidence of associations with WPPSI Z-scores. Sex was not associated with WPPSI Z-scores (table 3B).

The model for externalising behaviours Z-scores, with an R^2^=0.16, showed that feminine sex and higher sTfR (but not other iron biomarkers such as ferritin or haemoglobin) were associated with lower externalising behaviour Z-score. In contrast, higher externalising behaviour was associated with the early introduction of solid/semisolid foods (before 3 months), also with underweight in the first year, higher number of diarrhoeal episodes between 0 and 11 months, and higher Maternal Depression Score evaluated at 6 months. Bayley scores at 6 months showed weak evidence of a positive association with externalising behaviour Z-scores (table 3C).

## Discussion

Most children 5 years of age from the Naushahro Feroze, had executive functions and WPPSI intelligence tests below 0-Z scores. Also, the proportion of children with externalising behaviour was almost half of our sample. Different types of early stressors predicted developmental outcomes at 5 years. Among sociodemographic characteristics, the number of assets (a proxy for wealth) was associated with higher WPPSI Z-scores, whereas nutritional indicators (early time of introduction of solids and underweight in the first year) were associated with higher externalising behaviour at 5 years. Also, indicators of intestinal infections (AAT, and number of diarrhoeal episodes in the first year) were associated with lower WPPSI and higher externalising Z-scores. Other indicators such as the HOME score at 6 months and Bayley scores at 15 months predicted EF Z-scores at 5 years, and higher Maternal Depression Score predicted higher externalising behaviour at 5 years. Compared with other MAL-ED cohorts, children in this study were not achieving their full potential.

Instruments for evaluating child development might not adequately capture the complexity of social-emotional skills and behaviour when assessing children in disadvantaged populations. In the MAL-ED study, validation of instruments allowed to identify, for example, that the internalising behaviour component of the SDQ was not reliable in this setting. Also, some independent variables such as food insecurity, lower number of assets and poor diets, may indirectly reflect the impact of poverty on developmental outcomes, and we cannot infer specific causality. Others have shown that in-utero and neonatal factors such as preterm birth phenotypes^26^ and cranial growth^27^ are associated with neurodevelopment at 2 years, but in our study, data on gestational age at birth and head circumference were not available, which did not allow to explore associations of child development with these potential predictors. These limitations might had played a role in the low explanatory power of our models. Also, early determinants may be modified by experiences and conditions later in childhood, that may account for most of the variability of indices measured at 5 years. We acknowledge that the scope of the research could have benefitted from the inclusion of participants from other areas of Pakistan, in order to combat bias. However, the study design benefited from a rigorous process of data collection and a comprehensive range of predictors under difficult logistic conditions.

Research has shown that poverty can frame child neurodevelopment via the deprivation of stimuli and increased exposure to negative input, while having fewer protecting resources to overcome adverse circumstances.^28^ Poverty often coincides with food insecurity, which is known to affect child development through multiple paths, including a decreased quantity and quality of food and producing parental stress and anxiety.^29^ In our cohort, poverty (indicated by lower number of assets) was the main negative predictor of lower WPPSI intelligence test, mimicking the findings of the South Africa and Tanzania MAL-ED cohorts,^30^ and those of another study with 4-year-old children from Naushahro Feroze, where higher socioeconomic status was positively correlated with WPPSI-III.^31^ Although the differential impact of poverty and food insecurity on child development requires further research, our findings suggest that poverty-alleviation policies might be helpful to decrease impaired child development in deprived settings.

Among the MAL-ED measurements of infection/inflammation, faecal AAT concentrations, a sensitive marker of protein-losing enteropathy, were associated with lower WPPSI. In other countries of the MAL-ED study, AAT, was associated with lower growth indicated by WAZ,^32^ but not in Pakistan.^20^ To our knowledge, this is the first-time faecal AAT has been specifically associated with child intelligence measured by WPPSI. Moreover, an early start of solids/semisolids between 0 and 2 months and the number of diarrhoeal episodes were associated with higher externalising behaviour, possible related with evidence that contaminated complementary foods may be a source of protozoan and helminth parasites,^33^ and that diarrhoeal disease in the first 2 years of life is associated with cognitive impairment of children from developing settings.^34^ Our findings support the hypothesis that environmental enteropathy and its associated intestinal barrier disruption and impaired nutrient absorption, affects growth and neurodevelopment.^35^

Regarding sex variations in ECD, no morphological differences in brain development were found between male and female fetuses in healthy pregnancies from eight diverse study sites,^36^ but research has shown that emotion expressions differ by sex, boys expressing more externalising emotions (such as anger), whereas girls express more positive emotions but more internalising negative emotions (such as sadness and anxiety).^37^ This agrees with our findings, where externalising behaviour was higher in boys compared with girls. However, we did not find differences in EF or WPPSI Z-scores based on sex, and studies using these tests rarely report sex variation. Possible sex influence on developmental outcomes requires further research.

Child anthropometry has shown associations with developmental indicators in the MAL-ED cohort of Bangladesh, where stunting and underweight at 6–24 months were associated with poor ECD using Bayley scores.^38^ Also, a longitudinal analysis in seven of the eight MAL-ED countries (excluding Pakistan), found that 24-month Bayley scores were associated with summative weight from 18 to 24, but not with linear growth.^38^ We found that underweight during the first year was associated with higher externalising behaviour and presented weak evidence of an association with lower WPPSI Z-scores at 5 years. These findings complement information from Pakistan, where a positive correlation between higher HAZ and WPPSI-III has been previously reported.^31^

Among nutritional biomarkers, higher sTfR, an indicator of increased erythropoiesis in response to iron deficiency,^39^ was associated with lower externalising behaviour. We have previously reported that lower sTfR in the first year was associated with underweight and lower WAZ at 5 years in the same cohort.^20^ Given that sTfR is mainly produced by erythroid cells, which are decreased in protein-energy malnutrition,^40^ a low sTfR in presence of iron deficiency and anaemia may indicate early nutritional deficits, but this requires further investigation. Although other individual serum micronutrient indicators had weak associations or did not enter our models, our study does not rule out the possible influence of other macronutrient and micronutrient deficiencies on low scores of developmental indicators.

It has been demonstrated that infant cognitive development can predict long-term developmental outcomes, and failing developmental synapses in early childhood may be difficult to restore at a later age, as brain plasticity decreases.^3^ Consistent with this, we found that Bayley scores recorded at 15 months predicted higher EF Z-scores at 5 years, and that better quality of home environment at 6 months of age was associated with higher EF Z-scores. Moreover, externalising behaviour Z-scores was associated with higher score of maternal depression, whereas in the MAL-ED cohort of Bangladesh, Maternal Depression Score was not associated Bayley Z-scores at 6, 15 and 24 months of age.^38^ However, our findings agree with a comparable cohort of children where both HOME scores and SRQ scores had positive and negative correlations, respectively, with WPPSI-III.^31^ The possible influence of the home environment and maternal depression on adverse factors for child development are possible public health targets to address ECD.

## Conclusion

Indicators of executive function, intelligence and externalising behaviour were differentially associated with adverse conditions during the first year of life, commonly reflecting poverty and undernutrition. Our results highlight that early infancy factors might have a lasting effect on child development and that interventions aiming to address extreme poverty, food insecurity, early nutrition and decrease of infections in early childhood are needed to improve developmental outcomes of children in deprived settings.

## supplementary material

10.1136/bmjnph-2024-000900 online supplemental file 1

## Data Availability

Data are available upon reasonable request.

## References

[1] Daelmans B, Black MM, Lombardi J (2015). Effective interventions and strategies for improving early child development. BMJ.

[2] Silburn SR, Nutton G, Arney F (2011). The First 5 Years: Starting Early.

[3] Bhutta ZA, Bhavnani S, Betancourt TS (2023). Adverse childhood experiences and lifelong health. Nat Med.

[4] Loizillon A, Petrowski N, Britto P (2017). Development of the Early Childhood. Development Index in MICS surveys. MICS Meth Pap.

[5] Emerson E, Llewellyn G (2023). The prevalence of significant cognitive delay among 3- to 4-year-old children growing up in low- and middle-income countries: results from 126 nationally representative surveys undertaken in 73 countries. J Intellect Disabil Res.

[6] United Nations Children’s Fund (2023). The early childhood development index 2030: a new measure of early childhood development, unicef, new york, 2023.15.

[7] WHO (2023). Global scales for early development (GSED). https://www.who.int/publications/i/item/WHO-MSD-GSED-package-v1.0-2023.1.

[8] Murray-Kolb LE, Acosta AM, Burga RR (2018). Early childhood cognitive development is affected by interactions among illness, diet, enteropathogens and the home environment: findings from the MAL-ED birth cohort study. BMJ Glob Health.

[9] Obradović J, Finch JE, Portilla XA (2019). Early executive functioning in a global context: Developmental continuity and family protective factors. Dev Sci.

[10] Renaud F, Béliveau M-J, Akzam-Ouellette M-A (2022). Comparison of the Wechsler Preschool and Primary Scale of Intelligence-Third Edition and the Leiter-R Intellectual Assessments for Clinic-Referred Children. J Psychoeduc Assess.

[11] Stone LL, Otten R, Engels RCME (2010). Psychometric properties of the parent and teacher versions of the strengths and difficulties questionnaire for 4- to 12-year-olds: a review. Clin Child Fam Psychol Rev.

[12] Khan MA, Owais SS, Blacklock C (2017). Delivering integrated child development care in Pakistan: protocol for a clustered randomised trial. BJGP Open.

[13] McCormick BJJ, Richard SA, Caulfield LE (2019). Early Life Child Micronutrient Status, Maternal Reasoning, and a Nurturing Household Environment have Persistent Influences on Child Cognitive Development at Age 5 years: Results from MAL-ED. J Nutr.

[14] Malda M, Vijver F, Srinivasan K (2008). Adapting a cognitive test for a different culture: An illustration of qualitative procedures. Psychol Sci Q.

[15] Turab A, Soofi SB, Ahmed I (2014). Demographic, socioeconomic, and health characteristics of the MAL-ED network study site in rural Pakistan. Clin Infect Dis.

[16] Caulfield LE, Bose A, Chandyo RK (2014). Infant feeding practices, dietary adequacy, and micronutrient status measures in the MAL-ED study. Clin Infect Dis.

[17] Richard SA, Barrett LJ, Guerrant RL (2014). Disease surveillance methods used in the 8-site MAL-ED cohort study. Clin Infect Dis.

[18] Platts-Mills JA, Babji S, Bodhidatta L (2015). Pathogen-specific burdens of community diarrhoea in developing countries: a multisite birth cohort study (MAL-ED). Lancet Glob Health.

[19] Richard SA, McCormick BJJ, Murray-Kolb LE (2019). Enteric dysfunction and other factors associated with attained size at 5 years: MAL-ED birth cohort study findings. Am J Clin Nutr.

[20] González-Fernández D, Cousens S, Rizvi A (2023). Infections and nutrient deficiencies during infancy predict impaired growth at 5 years: Findings from the MAL-ED study in Pakistan. Front Nutr.

[21] WHO, UNICEF (2021). Indicators for Assessing Infant and Young Child Feeding Practices: Definitions and Measurement Methods.

[22] Pendergast LL, Schaefer BA, Murray-Kolb LE (2018). Assessing development across cultures: Invariance of the Bayley-III Scales Across Seven International MAL-ED sites. Sch Psychol Q.

[23] Jones PC, Pendergast LL, Schaefer BA (2017). Measuring home environments across cultures: Invariance of the HOME scale across eight international sites from the MAL-ED study. J Sch Psychol.

[24] Pendergast LL, Scharf RJ, Rasmussen ZA (2014). Postpartum depressive symptoms across time and place: structural invariance of the Self-Reporting Questionnaire among women from the international, multi-site MAL-ED study. J Affect Disord.

[25] Li C (2013). Little’s Test of Missing Completely at Random. The Stata Journal: Promoting communications on statistics and Stata.

[26] Villar J, Restrepo-Méndez MC, McGready R (2021). Association Between Preterm-Birth Phenotypes and Differential Morbidity, Growth, and Neurodevelopment at Age 2 Years: Results From the INTERBIO-21st Newborn Study. JAMA Pediatr.

[27] Villar J, Gunier RB, Tshivuila-Matala COO (2021). Fetal cranial growth trajectories are associated with growth and neurodevelopment at 2 years of age: INTERBIO-21st Fetal Study. Nat Med.

[28] Johnson SB, Riis JL, Noble KG (2016). State of the Art Review: Poverty and the Developing Brain. Pediatrics.

[29] Gallegos D, Eivers A, Sondergeld P (2021). Food Insecurity and Child Development: A State-of-the-Art Review. Int J Environ Res Public Health.

[30] Drago F, Scharf RJ, Maphula A (2020). Psychosocial and environmental determinants of child cognitive development in rural south africa and tanzania: findings from the mal-ed cohort. BMC Public Health.

[31] Rasheed MA, Pham S, Memon U (2018). Adaptation of the Wechsler Preschool and Primary Scale of Intelligence-III and lessons learned for evaluating intelligence in low-income settings. International Journal of School & Educational Psychology.

[32] Kosek M, Haque R, Lima A (2013). Fecal markers of intestinal inflammation and permeability associated with the subsequent acquisition of linear growth deficits in infants. Am J Trop Med Hyg.

[33] Palmieri JR, Meacham SL, Warehime J (2018). Relationships between the weaning period and the introduction of complementary foods in the transmission of gastrointestinal parasitic infections in children in Honduras. Res Rep Trop Med.

[34] John CC, Black MM, Nelson CA (2017). Neurodevelopment: The Impact of Nutrition and Inflammation During Early to Middle Childhood in Low-Resource Settings. Pediatrics.

[35] Bhutta ZA, Guerrant RL, Nelson CA (2017). Neurodevelopment, Nutrition, and Inflammation: The Evolving Global Child Health Landscape. Pediatrics.

[36] Namburete AIL, Papież BW, Fernandes M (2023). Normative spatiotemporal fetal brain maturation with satisfactory development at 2 years. Nature New Biol.

[37] Chaplin TM, Aldao A (2011). Gender Differences in Emotion Expression in Children: A Meta-Analytic Review. Psychol Bull.

[38] Nahar B, Hossain M, Mahfuz M (2020). Early childhood development and stunting: Findings from the MAL-ED birth cohort study in Bangladesh. Matern Child Nutr.

[39] Beguin Y (2003). Soluble transferrin receptor for the evaluation of erythropoiesis and iron status. Clin Chim Acta.

[40] Borelli P, Blatt S, Pereira J (2007). Reduction of erythroid progenitors in protein-energy malnutrition. Br J Nutr.

