# Supplementary material for: Early life adverse environmental, nutrition and infection factors are associated with lower developmental scores in Pakistani children at 5 years: a cohort study
Source: BMJ Nutr Prev Health. 2024 Nov 7;7(2):e000900. doi: 10.1136/bmjnph-2024-000900 (PMC11773647; doi:10.1136/bmjnph-2024-000900)
Supplement: online supplemental file 1 [file bmjnph-7-2-s001.pdf]

Supplementary material - Early life adverse environmental, nutrition and infection factors are associated with lower developmental scores in Pakistani children at 5 years: a cohort study.

Table S1. Quality assessment of dependent variables recorded at 5 years of age.

**General quality assessment**	Quality of data collection was ensured by training of personnel, expert analysis of videorecording (10-20% tasks administrations), with feedback to field researchers, and by expert visits to the site for observing data collection procedures. Data quality was assessed using: (a) preliminary analyses (e.g., descriptive statistics, missing data handling, removal of zero variance items and extreme outliers), (b) exploratory factor analysis (EFA), (c) confirmatory factor analysis (CFA), (d) multiple indicator multiple cause modeling (MIMIC), and (e) reliability analyses. Best practice procedures were followed while conducting EFA (1). A detailed review of the MIMIC modeling approaches employed by this research group for all measures can be found elsewhere (2, 3).
**Executive Functions test (EF)**	Composite scores were first created for each of the subscales by adding the total number of correct items. For the calculation of Z-scores, data from Nepal was excluded, as data did not meet basic assumptions for factor analyses. The validity of scores at six of the seven sites (Bangladesh, Brazil, India, Pakistan, South Africa, and Tanzania) was supported, and the validity of scores appeared to be comparable across sites. However, the overall reliability of the executive functioning factor among MAL-ED countries was low (α=0.57), indicating that these scores should be interpreted with caution.
**Wechsler Preschool and Primary Scale – Third Edition (WPPSI-III)**	EFA and CFA determined that four subscales had the potential to yield meaningful scores in the context of cross-site analyses: Block Design, Matrix Reasoning, Picture Concepts, and Picture Completion. Using MIMIC modeling, it was found that overall, for the Block Design, Picture Completion, and Matrix Reasoning subtests, most items were free of bias at the item level. Conversely, for Picture Concepts, a substantial number of items displayed significant and meaningful differential item functioning. Therefore, the Picture Concepts subscale was removed from subsequent analyses. The final, higher-order model, including items from the Block Design (scored 0-15), Picture Concepts (scored 0-9), and Matrix Reasoning (scored 0-15) subtests, was submitted for a final analysis using CFA. The higher-order model was a good fit to the data. Further, the Perceptual Reasoning factor demonstrated good reliability for the entire sample (α=0.83) and minimally adequate reliability (for research purposes) at the site level with estimates equals to 0.62 in Pakistan.
**Strengths and difficulties questinnaire (SDQ)**	The recommended SDQ factors vary widely between studies and there is substantial disagreement between researchers regarding which subscale scores should be used. In this study, the model that yielded the best fit statistics (RMSEA=.063, CFI=.90, X^2=987.53, $p<.001$) was the three-factor model including Internalizing, Externalizing, and Prosocial factors. Overall, psychometric analyses yielded insufficient support for the Internalizing Symptoms factor (Factor I) with low reliability and a high rate of differential item functioning for most items. The prosocial factor (Factor III) had low reliability in MIMIC. For factor II (Externalizing Symptoms), reliability was adequate, with α=0.73.

Table S2. Independent variables recorded between 0-11 months of age.

Independent variables			Assessment method
**Distal predictors**	Socio-demographic characteristics	Number of assets (0-8): mattress, chair, table, tv, refrigerator, bank account, separated kitchen, < 2 people per room. Positive responses for each of these get a score of 1 while negative responses get 0., income (continuous variable), maternal education (years), having improved sanitation, number of people per room.	Interviews performed an average of 1.6 times in the first year
	Food insecurity category	Coded as 0: no food insecurity, 1: mild food insecurity, 2: moderate/severe food insecurity	
**Intermediate predictors**	Breastfeeding and complementary feeding practices	WHO's indicators for assessing infant and young child feeding practices (4). Among the following, variables were considered for analysis if at least 10% of children were exposed:<br>- children exclusively breastfed under 6 months.<br>- mixed milk feeding under six months.<br>- continued breastfeeding, 6-11 months.<br>- received solid/semisolid foods 6-8 months.<br>- Minimal dietary diversity 6-11 months (yes/no): Defined as consumed foods and beverages from at least five out of eight food groups during the previous day [1]<br>- Minimum meal frequency (yes/no, according to their age period 6-8 or 9-11 months) [2]<br>- Received animal-source foods 6-11 months.<br>- Received fruits and vegetables 6-11 months.<br>- Received unhealthy foods 6-11 months (sugar/ commercially available foods)	Twelve (one per month) 24-h recalls were performed.<br>Average of 350 observations per child during the first year (5).<br>Average intakes between 0-5 and 6-11 months were used for analyses.
	Illnesses during the first year of life	- mean days per month of illnesses in the first year of life<br>- days presenting acute low respiratory infection (ALRI)<br>- number of diarrheal episodes<br>- mean score of diarrhea (comprises duration, intensity, presence of fever, vomit, dehydration, hospitalization due to diarrhea (6)).	Information was recorded twice a week during the first year. Average of 351 observations per child.<br>Caregivers were asked whether the child had been ill or had experienced symptoms each day since the last visit (7)
	Pathogens	Detected pathogens (Adenovirus, Astrovirus, Norovirus, Rotavirus, *Aeromonas, E. coli,*	Stool samples were collected on average

[1] WHO defined food groups: 1. breast milk; 2. grains, roots, tubers and plantains; 3. pulses (beans, peas, lentils), nuts and seeds; 4. dairy products (milk, infant formula, yogurt, cheese); 5. flesh foods (meat, fish, poultry, organ meats); 6. eggs; 7. vitamin-A rich fruits and vegetables; and 8. other fruits and vegetables.

[2] Minimum meal frequency defined as yes if: breastfed infants 6‑8 months were provided complementary foods 2‑3 times per day; breastfed children 9‑23 months were provided complementary foods 3‑4 times per day with additional nutritious snacks offered 1‑2 times per day; or non-breastfed children increase previous recommendation to 4‑5 meals per day

		Campylobacter, Vibrio, Salmonella, Shigella, Entamoeba hystolitica, Giardia, and *Cryptosporidium*) were grouped as infection by viruses, bacteria, and protozoa accordingly, and were evaluated in their association with diarrhea as: 0: absent, 1: present/no diarrhea, 2: present+diarrhea.	once a month during the first year of life
	Fecal biomarkers (8)	- Myeloperoxidase (indicator of neutrophil activation)<br>- Neopterin (indicates T-helper cell activity)<br>- Alpha-1 antitrypsin (biomarker of protein-loss enteropathy)	
	Anthropometry 0-11 months	Means of:<br>- Weight-for-age (WAZ) / underweight<br>- Length-for-age (LAZ) / stunting<br>- Weight for length (WLZ) / wasting<br>- Head circumference (HCAZ) / low HCAZ for age<br>Longitudinal variables (Z-scores) or binary variables indicating undernutrition were used for analyses, when measures fell under -2 Z-scores	Average of 12 measurements of weight, and 8 of height and head circumference per child were taken in the first year
	Nutrient deficiencies and inflammation (9-12)	- anemia (hemoglobin <11 g/dL),<br>- low ferritin <12 ug/L,<br>- elevated serum transferrin receptor (sTfR) >67 mg/L,<br>- vitamin A deficiency <20 ug/dL<br>- zinc deficiency <10.07,<br>- low urinary iodine <100 umol/L,<br>- alpha-1 glycoprotein (AGP) >100 g/dL	Blood and urine samples for nutritional indicators were taken once during the first year of age (mean age of 6 months, range 5-8 months), and were available for a subsample of children
**Proximal predictors**	Development (13)	- Bayley scores	Tests performed at 6 and 15 months of age[3]
	Home environment (14, 15)	- Home Observation for Measurement of the Environment (HOME) Inventory.[4]	Evaluations performed at 6 months of age
	Maternal depression (16)	- Maternal depression score – SRQ (3).[5]	

[3] Bayley scores usually include 5 individually administered subscales (cognitive, fine motor, gross motor, receptive language and expressive language), and 2 parent-completed rating scales (social-emotional and adaptive behaviour) (15). For the MAL-ED cohort, the adaptive behavior questionnaire was not collected, due to its overlap with items on the temperament scale used in the study, and to the time commitment required for its application (2). To identify the factor structure of the Bayley-II scores, exploratory and confirmatory factor analyses were performed, followed by reliability and item response theory analyses, and for assessing invariance, multiple-indicator, multiple-cause modeling were applied (16). For the Pakistan cohort, findings supported the validity, but not invariance, of language, cognitive and motor scores (16).

[4] Evaluated behaviors at 6 months of age included scores on emotional and verbal responsivity of caregiver, avoidance of restriction and punishment, caregiver's promotion of child development, organization of physical and temporal environment, provision of appropriate play materials, opportunities for variety in daily stimulation and cleanliness of child subscales (13).

[5] Items included: Trouble thinking clearly, feeling nervous/tense/worried, feeling worthless, thinking of ending life, crying more often, loss of interest, feeling unhappy, difficulty enjoying activities, tiring easily, hands shake, daily work suffering, unable to play a useful part in life, difficulty making decisions, always tired, and easily frightened, loss of appetite. Somatic symptoms usually included in the

Table S3. Percentage of health indicators recorded as (A) Blood/urine biomarkers, (B) Presence of illnesses, (C) environmental enteropathy indicators and (D) intestinal pathogens during the first year of life.

Sample of 204 children unless otherwise specified.

**A. Abnormal nutritional serum/urine indicators, 0-11 months**	**n**	**%**
Hemoglobin <11 g/dL (n=201)	142	70.6
Alpha glycoprotein >100 mg/dL (n=197)	74	37.6
Ferritin <12 ug/L or >30 ug/L in children with elevated AGP (n=163)	63	38.6
Retinol <20 ug/dL (n=187)	101	54.0
sTfR >6.67 mg/L (n=198)	11	5.6
Zinc <0.07 umol/L (n=192)	161	83.8
Urinary iodine <100 ug/L (n=198)	24	12.1
**B. Illnesses 0-11 months**	**Median**	**IQR**
Mean days/month presenting any illness	24	18, 27
Days presenting acute low respiratory infection	7	3, 13
Number of diarrheal episodes	7	4, 10
**C. Mean environmental enteropathy indicators**		
Myeloperoxidase, ng/mL	2.4	1.2, 8.0
Neopterin, nmol/L	2.0	1.1, 4.1
Alpha-1 antitrypsin, µmol/L	0.9	0.3, 1.7
**D. Pathogens in stools (positive in any sample taken during the first year)**	**Present, no diarrhea**	**Present +diarrhea**
Viruses (Astrovirus, Norovirus, Rotavirus, Adenovirus)	47 (23.0%)	123 (60.3%)
Bacteria (*Aeromonas, E. coli, Campylobacter, Salmonella, Shigella, Vibrio*)	41 (20.1%)	163 (79.9%)
Protozoa (*Cryptosporidium, E. histolytica, Giardia*)	82 (40.2%)	107 (52.4%)

Average samples per child during the first year: (A) 1, (B) 351, (C) 200, (D) 12.

SRQ (headaches, stomachaches, digestive difficulties, and sleep difficulties) were excluded from analyses, given that they may be present in post-partum mothers in a context of multiple infections, and may not necessarily reflect internalizing symptoms. Each question scored as 0 if the answer was negative or 1 if the answer was positive (for a total of 16 points) (3).

Figure S1. Histograms of developmental outcomes' Z-scores

Dashed lines show a Z-score=0. Proportion of children with developmental scales below 0-Z-scores for Z-EF and Z-WPPSI, and Z-externalizing behavior above 0-Z scores are shown.

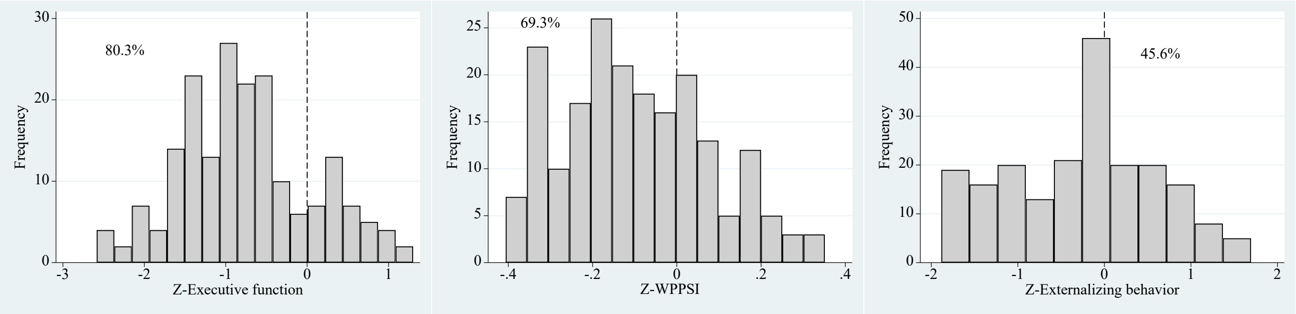

References

1. Fabrigar LR, MacCallum RC, Wegener DT, et al. Evaluating the use of exploratory factor analysis in psychological research. *Psychological Methods* 1999;4(3):272-99. doi: 10.1037/1082-989X.4.3.272

2. Murray-Kolb LE, Rasmussen ZA, Scharf RJ, et al. The MAL-ED cohort study: Methods and lessons learned when assessing early child development and caregiving mediators in infants and young children in 8 low-and middle-income countries. *Clinical Infectious Diseases* 2014;59(Suppl 4):S261-S72. doi: 10.1093/cid/ciu437

3. Pendergast LL, Scharf RJ, Rasmussen ZA, et al. Postpartum depressive symptoms across time and place: Structural invariance of the Self-Reporting Questionnaire among women from the international, multi-site MAL-ED study. *Journal of Affective Disorders* 2014;167(215):178-86. doi: 10.1016/j.jad.2014.05.039

4. WHO, UNICEF. Indicators for Assessing Infant and Young Child Feeding Practices2021.

5. Antiporta DA, Ambikapathi R, Bose A, et al. Micronutrient intake and the probability of nutrient adequacy among children 9-24 months of age: Results from the MAL-ED birth cohort study. *Public health nutrition* 2021;24(9):2592-602. doi: 10.1017/S1368980020000877

6. Lee GO, Richard SA, Kang G, et al. A comparison of diarrheal severity scores in the MAL-ED multisite community-based cohort study. *Journal of pediatric gastroenterology and nutrition* 2016;63(5):466-73. doi: 10.1097/MPG.0000000000001286

7. Richard SA, Barrett LJ, Guerrant RL, et al. Disease surveillance methods used in the 8-site MAL-ED cohort study. *Clinical Infectious Diseases* 2014;59(Suppl 4):S220-S24. doi: 10.1093/cid/ciu435

8. McCormick BJJ, Murray-Kolb LE, Lee GO, et al. Intestinal permeability and inflammation mediate the association between nutrient density of complementary foods and biochemical measures of micronutrient status in young children: Results from the MAL-ED study. *American Journal of Clinical Nutrition* 2019;110(4):1015-25. doi: 10.1093/ajcn/nqz151

9. WHO. Serum retinol concentrations for determining the prevalence of vitamin A deficiency in populations. [online]. Geneva: World Health Organization; 2011 https://www.who.int/publications/i/item/WHO-NMH-NHD-MNM-11.3 (accessed December 10, 2023).

10. WHO. WHO guideline on use of ferritin concentrations to assess iron status in individuals and populations [Internet]. World Health Organization; 2020 https://www.who.int/publications/i/item/9789240000124 (accessed December 11, 2023).

11. Willoughby JL, Bowen CN. Zinc deficiency and toxicity in pediatric practice. *Curr Opin Pediatr* 2014;26(5):579-84. doi: 10.1097/MOP.0000000000000132

12. WHO. Haemoglobin Concentrations for the Diagnosis of Anaemia and Assessment of Severity. 2011. http://www.who.int/vmnis/indicators/haemoglobin.pdf (accessed 2 Dec 2016).

13. Bayley N. Bayley Scales of Infant and Toddler Development 3rd Edition: Screening Test Manual. San Antonio, TX: Harcourt Assessment, Inc 2006.

14. Jones PC, Pendergast LL, Schaefer BA, et al. Measuring home environments across cultures: Invariance of the HOME scale across eight international sites from the MAL-ED study. *Journal of School Psychology* 2017;64(April):109-27. doi: 10.1016/j.jsp.2017.06.001

15. Alam MA, Richard SA, Fahim SM, et al. Impact of early-onset persistent stunting on cognitive development at 5 years of age: Results from a multi-country cohort study. *PLoS ONE* 2020;15(1):1-16. doi: 10.1371/journal.pone.0227839

16. Pendergast LL, Schaefer BA, Murray-Kolb LE, et al. Assessing development across cultures: Invariance of the Bayley-III Scales Across Seven International MAL-ED sites. *School Psychology Quarterly* 2018;33(4):604-14. doi: 10.1037/spq0000264
